# Trends and Prospects of Biometrics: From Sensing to Perception and Cognition

**DOI:** 10.3390/s26092571

**Published:** 2026-04-22

**Authors:** Zhicheng Cao, Natalia Schmid, Liaojun Pang

**Affiliations:** 1 Molecular and Neuroimaging Engineering Research Center of Ministry of Education, School of Life Science and Technology, Xidian University, Xi’an 710126, China; 2 Lane Department of Computer Science and Electrical Engineering, West Virginia University, Morgantown, WV 26506, USA; natalia.schmid@mail.wvu.edu

**Keywords:** biometrics, sensor, trends, artificial intelligence, modality

## Abstract

Biometrics technology is undergoing a paradigm shift from static single-modal authentication to continuous multimodal sensing, combined with higher-performing algorithms powered by new deep learning techniques. This editorial reviews cutting-edge advancements and trends in the field of biometrics in four dimensions—novel sensors, modalities, algorithms, and equipment—as well as summarizes the contributions to this Special Issue, “New Trends in Biometric Sensing and Information Processing” by grouping them into the corresponding aspects of breakthroughs in this field.

## 1. Introduction

In the era of digitization and informatization in modern society, accurate, convenient, and secure identity authentication has become an infrastructure necessity. Traditional authentication methods based on passwords, documents, or PIN codes, can no longer meet the need for increasingly complex cybersecurity due to their inherent flaws and issues, such as forgetfulness, theft, and forgery [1,2,3]. Biometrics technology, which verifies identity by measuring an individual’s unique physiological or behavioral traits [4], has transitioned from an auxiliary tool to a mainstream authentication solution owing to its innate indivisibility and resistance to replication.

For instance, as one of the pioneering biometrics techniques, fingerprint recognition [5] was widely used in criminal identification in the early 20th century due to its “uniqueness” and “invariance”. However, fingerprint comparison relies entirely on manual visual inspection and expert experience, which is inefficient and was difficult to popularize. In the second half of the 20th century, with breakthroughs in computer technology and optical imaging, the era of automated recognition began. Also known as an Automatic Fingerprint Identification System (AFIS), fingerprint recognition has taken the lead in automation. Automatic feature point extraction and matching algorithms such as FingerCode [6], the Tico Descriptor [7], MCC [8] have enabled fingerprint recognition to transition from police to civilian use. Sensor techniques for fingerprints [9] have evolved from optical fingerprint sensors [10] to semiconductor fingerprint sensors (such as capacitive [11], pressure-based [12], and thermal [13] sensors) and then to ultrasound fingerprint sensors [14], which have high imaging quality and great potential for under-screen fingerprint recognition on mobile phones.

Facial recognition [15] emerged as an alternative to fingerprint recognition due to its advantages such as ease of acquisition, convenience, and user-friendly experience. Early facial recognition algorithms were based on geometric features such as facial spacing [16]. Later, photometric methods such as EigenFace (or PCA) [17] and LBP [18] gradually replaced geometric methods. Although limited by lighting and posture, photometric methods became promising modalities due to their non-contact nature. As another popular biometric modality, iris recognition [19] stands out due to its extremely high accuracy and anti-counterfeiting capabilities, and its algorithm utilizes the rich texture structure of the iris. Iris recognition has thus become the first choice for high-security scenarios. Other modalities such as gait recognition [20] have filled the gap in remote recognition, which uses dynamic features to play a unique role in security monitoring.

However, the field of biometrics is standing at a technological turning point. Single-modal biometric systems (such as relying solely on fingerprints or faces) face limitations in dealing with fluctuations in data quality, environmental changes, and malicious attacks. Additionally, the miniaturization of sensors, advancements in flexible electronic technology, breakthroughs in deep learning algorithms, and the popularity of wearable devices have jointly spurred a new paradigm in biometrics. The core features of this paradigm can be summarized in four transformations: from rigid sensors to flexible wearable sensors, from static physiological features to live and dynamic signals, from manual feature design to the automatic representation of large models, and from single-function devices to integrated multifunctional devices.

## 2. Current Trends in the Field

### 2.1. New Biometric Sensors

Sensing technology is the first aspect for biometrics, and sensing performance directly determines the quality and potential of the raw data. In recent years, breakthroughs in materials science and micro/nano-manufacturing have been redefining the possible forms of sensors [21].

Traditional rigid sensors face two major challenges in wearable scenarios: limited fit of human body curves and degradation in signal quality caused by motion artifacts. The BioTopo Life Pocket wearable sensor, based on topologically protected flexible metasurfaces ad proposed by a research team from Southeast University, China, provides a breakthrough solution [22]. This sensor utilizes the discrete structural characteristics of topological metasurfaces to ensure the stable transmission of electromagnetic waves in bending, folding, or local fracture states. An experiment showed that the sensor maintains high-bandpass characteristics at seven folding angles ranging from 30° to 150°, and the transmission efficiency is always high. More importantly, by capturing the periodic modulation in electromagnetic waves as well as in human heart and lung movements, the sensor can synchronously monitor vital signs and verify individual identity in various states such as when lying down, moving, sitting, and standing. The accuracy of recognizing individuals in the supine position is 98.06%. This achievement marks a leapinof wearable sensors from usable to durable and applicable.

In another aspect of biometric sensor innovation, the emergence of all-organic transistor sensors has opened up new dimensions for flexible biometric sensors. The world’s first all-organic–transistor-active–matrix fingerprint sensor was developed in collaboration between Smartkem and Shanghai Jiao tong University, which achieved high-sensitivity fingerprint acquisition on curved surfaces [23]. The intrinsic properties of organic materials enable them to support multi-wavelength dynamic imaging, capture subcutaneous blood flow signals or sweat secretion dynamics, and effectively distinguish live fingerprints from silicone counterfeit products, which has long been a security issue with fingerprint recognition.

### 2.2. New Biometric Modalities

Bioelectrical signals such as electrocardiograms (ECGs) are becoming the core in multimodal recognition systems due to their inherent biological properties and individual specificity. A systematic review covering 80 studies from 2018 to 2023 showed that the average accuracy of ECG-based identity authentication systems is 98.6%, whereas the accuracy of multimodal fusion systems (such as ECG + fingerprint + iris) generally exceeds 99%. The advantages of ECG are, first, the signal originating from cardiac electrophysiological activity and can only be collected in a living state, naturally resisting forgery attacks; and second, the morphology of ECG being determined by the individual’s cardiac anatomy and electrophysiological characteristics, which are stable in the long-term.

Eye tracking, as an emerging representative of behavioral biology, is based on the theory that an individual’s visual search strategy, gaze duration distribution, and scanning path are jointly regulated by cognitive processes and neural mechanisms, making them difficult to externally. imitate Inspired by foraging theory, Srivastava and Patel proposed an eye movement modeling framework based on the Ornstein–Uhlenbeck process, which extracts individual specific stochastic differential equation parameters through Bayesian estimation and then integrates RNN and XGBoost for classification [24]. On the FIFA eye tracking dataset, the system achieved an average accuracy of 94% and an AUC of 98.97%, while maintaining an error rate of 4.0%. This method is a breakthrough: transitioning the use of eye movements from simple statistical features such as gaze duration to dynamic models that incorporate cognitive structures, enhancing the interpretability and anti-interference ability of these features.

In recent years, breakthroughs have also been achieved in the field of 3D face recognition. Traditional 2D face recognition is sensitive to lighting, pose, occlusion, and spoofing. Three-dimensional multimodal hybrid models achieved an accuracy of 99.74% in face validation under a strict false acceptance rate of 0.001 [25]. In terms of liveness detection (also known as face antispoofing), researchers developed a multimodal liveness detection algorithm that integrates infrared structured light, 3D depth maps, and micro-expression kinematics to address advanced forgery attacks such as 3D masks, head models, and screen remakes [26].

Another new modality in biometrics (specific for face recognition) has also drawn attention. This modality considers the infrared (IR) bands of light including near infrared (NIR), mid-wave infrared (MWIR), and long-wave infrared (LWIR) [27,28]. Unlike visible light, IR can be used to visualize dark and harsh atmospheric conditions, which are common in many real-world applications, such as during night-time surveillance, law enforcement, and military operations. The current mainstream methods for cross-spectral face recognition can be divided into two categories: cross-spectral feature alignment and cross-spectral image synthesis [29,30,31,32,33,34]. The former methods involve mapping visible light and IR images to a common feature subspace and reducing modal differences through metric learning. The latter methods are based on generative models such as GANs to synthesize IR images into “pseudo visible light” images.

### 2.3. New Algorithms: Deep Learning and Mutimodal Fusion

Algorithms are the “brain” of biometric systems. In recent years, deep learning has largely been applied to this field and has gradually replaced traditional algorithms based on hand-crafted operators and non-deep-learning methods. Additionally, given their rapid development, deep learning algorithms are also shifting from convolutional neural networks (CNNs) to Transformer architectures (such as ViT) [35]. Researchers have also focused on multimodal fusion strategies.

The successful application of Vision Transformer (ViT) [36] and its variant Swin Transformer [37] in biometrics marks a shift from local feature extraction to global relationship modeling in this field. Traditional CNN is limited by the size of the receptive field, which hinders the capturing of long-range dependencies in biometric features such as iris texture or fingerprint ridges. In contrast, the self-attention mechanism of Transformer can model the association between any two points in a feature map, which is particularly important for handling deformed, occluded, or partially missing biometric samples. Swin Transformer achieves multiscale feature extraction while maintaining computational efficiency by introducing hierarchical design and moving window mechanism. For instance, in iris recognition tasks, Swin Transformer outperformed the classical Daugman algorithm and traditional CNN methods [38]. In fingerprint recognition, applying the attention mechanism of Transformer effectively enhanced the representation ability of fine features [39].

Multimodal fusion is the key to increasing system robustness. Depending on the level of fusion, multimodal fusion can be divided into sensor, feature, score, and decision levels [40]. In recent years, research showed that feature-level fusion is increasingly favored due to its ability to preserve the rich information in original features [41], but it also faces the challenges with dimensionality and feature alignment.

At the fusion level, researchers [42] integrated iris, facial, and finger vein feature, demonstrating the effectiveness of multimodal feature fusion based on deep learning methods. The authors used a pre-trained CNN (such as ResNet and FaceNet) and ViT for feature extraction and flexibly combined different modalities through a score-level fusion strategy, achieving a recognition accuracy of 99%.

### 2.4. New Equipment: Wearable and Edge Intelligence

Advancements in sensing materials, biological modalities, and algorithms have culminated in the innovation of terminal devices/equipment. Biometric systems are transitioning from specialized acquisition terminals that are usually non-mobile or non-wearable, such as fingerprint scanners and iris cameras, to everyday mobile or wearable devices.

A smart photonic wristband designed by a multinational research team [43] represents the forefront of this field. This wristband integrates cardiovascular and pulmonary function monitoring (such as respiratory rate (RR), heart rate (HR), and blood pressure (BP)) as well as biometric identification functions that traditionally require bulky medical equipment into a wristband device. The wristband performs two functions: health management and safety authentication, which are technically possible at the technical level due to the miniaturization, flexibility, and end-to-end integration of Solaris polymer optical fiber (SPOF) sensors with deep learning algorithms.

Edge intelligence and devices have received increasing attention in recent years due to their advantages in real-time processing, on-device decision-making, reduced latency, enhanced privacy, and optimized resource use [44,45]. However, the traditional architectures of visual chips experience serious latency and energy consumption overhead. Processing-in-memory (PIM) [46] and in-sensor computing (ISC) are promising computing architecture solutions for future edge intelligence. Inspired by biological visual systems, a research team from Tsinghua University designed and fabricated a new type of double-layer oxide photo memory resistor. Based on this, a prototype of a fully in-sensor reservoir computing system based on all-optical photo memory (OEM) resistors was constructed, achieving an accuracy of 91.2% with extremely low energy consumption in human motion recognition tasks [47].

## 3. Overview of Published Papers

This Special Issue entitled “New Trends in Biometric Sensing and Information Processing” aims to collate and disseminate state-of-the-art research advances in this field in new sensors, modalities, algorithms, systems, and applications. The scope of this Special Issue includes emerging trends and recent advances in sensing-related technologies in biometrics application. The following are the main research and development topics in this Special Issue:

- New sensor technologies with applications in biometrics;
- Wireless sensor networks for biometrics;
- Internet of Things in biometrics applications;
- New imaging modalities and technologies in biometrics;
- New information processing methods in biometrics;
- New machine learning algorithms in biometrics;
- New systems and instruments in biometrics;
- New applications of biometric sensing technologies;
- Other emerging sensing technologies in biometrics.

These contributions of the accepted papers are outlined in more detail below. Contributions 1 and 5 focus on the issue of biometric security and antispoofing. In Contribution 1, the authors developed a light-weighted fingerprint liveness detection network based on CycleGan and an improved ResNet with a multihead self-attention mechanism. Experiments on multiple datasets showed that the proposed method achieved accurate results. In Contribution 5, the authors introduced a detection network for deep forgery face images, where a GANis used to capture local forgery and artifacts, and Transformers are used to model global dependencies and predict anomalies in forged images using the frequency domain and noise information.

Contributions 2 and 3 are dedicated to the topic of face recognition, with the former dealing with 3D face generation and the latter studying the unusually used infrared band. The authors of Contribution 2 generated 3D synthetic face data using game engines and then conducted face recognition experiments and analysis. Future directions for improving synthetic biometric data generation and its impact on advancing biometrics research were also discussed.

The authors of Contribution 3 studied hyperspectral face recognition using a deep-learning-based fusion model with four modules: a pre-fusion scheme, a Siamese encoder with bi-scope residual dense learning, a feedback-style decoder, and a recognition-oriented composite loss function. Te experimental results proved the advantage of hyperspectral face recognition over face recognition using single light bands and justified the superiority of the proposed model.

Contribution 4 is dedicated to the topic of speech recognition of a minor language, Tibetan. Connectionist Temporal Classification (CTC) with maximum entropy optimization and the Probabilistic Sparse Attention Mechanism were integrated for automatic alignment and attention weight extraction. The experimental results showed that the model produced fewer errors than prior methods on self-constructed and open-source datasets.

Contributions 6 and 8 are related to eye-movement-based biometrics. The former study investigated how to use eye trackers to measure and adjust the salience of alarms using three methods with the gaze-based acknowledgement of alarms that estimate operator anticipation. The latter study examined two different methods for eye-movement-based biometric identification: eye movement dynamics and statistical values. Long short-term memory (LSTM) and dense networks were used in the identification experiments, with the first method being more accurate than the second method.

Contribution 7 is a review of the impact of biometric surveillance on reducing violent crime. The authors summarized the current state of biometric surveillance systems and analyzed the need of frameworks that ensure improvements in violent crime prevention while providing moral accountability and equitable implementation in diverse communities.

Contributions 9 and 10 detail studies on bioelectrical-signal-based biometrics. Contribution 8 discussed how to authenticate EEG by extracting power spectral density (PSD) features and using deep learning classifiers. The findings of this study support the feasibility of EEG-based biometrics as a secure, noninvasive authentication modality. Contribution 9 proposes a framework for EEG biometrics that integrates 1D and 2D EEG representations through collaborative embedding, dimensional attention weight learning, and projection matrix learning. The experimental results showed that their method is the state of the art.

Focused on iris biometrics, the authors of Contribution 11 developed a quantum-inspired deep model built on a customized ResNet-18 architecture augmented with a quanvolutional layer. Identification and authentication experiments on multiple iris datasets showed that quantum-inspired modifications are practical and scalable for enhancing the discriminative capacity of ResNet.

## 4. Conclusions

This Special Issue comprises eleven selected papers that explore various topics related to sensing and information processing in the area of biometrics research, covering several representative topics and cutting-edge technological advancements and promising applications. The goal of this collection is to provide the excellent opportunity to bring together the international biometrics community and to provide valuable highlights and references for readers from academia and industry for understanding the current trends, existing challenges, and future research directions in biometrics. Lastly, I would like to extend my deepest gratitude to all the authors who contributed to this Special Issue as well as to the reviewers and administrative editors who spent valuable time to ensure the quality and success of this Special Issue.

## Data Availability

No new data were created or analyzed in this study. Data sharing is not applicable to this article.

## Abbreviations

The following abbreviations are used in this manuscript:

AI	Artificial Intelligence
AFIS	Automatic Fingerprint Identification Systems
AOT	All-Organic-Transistor
AUC	Area Under Curve
BP	Blood Pressure
CNN	Convolutional Neural Network
CTC	Connectionist Temporal Classification
ECG	Electrocardiography
EEG	Electroencephalography
FIFA	Fédération Internationale de Football Association
GANs	Generative Adversarial Networks
HR	Heart Rate
ISC	In-Sensor computing
LBP	Local Binary Pattern
LSTM	Long Short-Term Memory
LWIR	Long-Wave Infrared
MCC	Minutia Cylinder-Code
MWIR	Mid-Wave Infrared
NIR	Near Infrared
OEM	Optical Photo Memory
PIM	Processing In Memory
PCA	Principal Component Analysis
PSD	power spectral density
RR	Respiratory Rate
SPOF	Solaris Polymer Optical Fiber
RNN	Recurrent Neural Network
ViT	Visual Transformer
XGBoost	eXtreme Gradient Boosting
